# Lauryl gallate promotes platelet activation and thrombus formation: a promising application to stop bleeding

**DOI:** 10.1042/CS20257213

**Published:** 2025-12-18

**Authors:** Basma Hadjkacem, Cédric Garcia, Jennifer Series, Véronique Pons, Asma Mahmoudi, Céline Gales, Sophie Voisin, Agnès Ribes, Marie-Pierre Gratacap, Ali Gargouri, Bernard Payrastre

**Affiliations:** 1 Laboratory of Molecular Biotechnology of Eucaryotes (LR15CBS02), Biotechnology Center of Sfax, University of Sfax; 2 Department of Life Sciences, Faculty of Sciences of Gafsa, University of Gafsa, Gafsa, Tunisia; 3 INSERM, UMR1297 and Université Toulouse 3, Institut des Maladies Métaboliques et Cardiovasculaires (I2MC), Toulouse, France; 4 Laboratoire d’Hématologie, Centre Hospitalier de Toulouse, Toulouse, France; 5 Laboratory of Environmental Bioprocesses, Center of Biotechnology of Sfax, P.O. Box 1177, 3018 Sfax, Tunisia

**Keywords:** antihemorrhagic potential, platelet signaling, hemostatic agent, lauryl gallate, platelet aggregation

## Abstract

Lauryl gallate (LG, E312) is an antioxidant with hydrophobic properties that contribute to its activity by increasing affinity for membranes and acting on the lipid phase transition and likely the lateral membrane organization. Here, we show that LG at a low concentration has the ability to spontaneously induce washed human platelet shape change, filopodia emission, granule secretion, and aggregation. LG was able to activate intracellular signaling pathways, including Akt, p38MAP kinase, inositol phosphate, and calcium responses, as well as to trigger cyclic adenosine monophosphate decrease and αIIbβ3 integrin activation. LG significantly potentiated platelet aggregation induced by low concentrations of agonists, and the addition of low doses of LG to human blood strongly increased the platelet thrombotic response under arterial flow on a collagen matrix. Morphological analysis by scanning electron microscopy indicated that contrary to low doses, high concentrations of LG induced dramatic platelet membrane modifications associated with calcium influx, lactate dehydrogenase leakage, and a slow platelet aggregation response. Interestingly, a local flash application of LG efficiently decreased the tail bleeding time in rats. LG action was rapid and significantly more efficient than tranexamic acid, an antifibrinolytic agent, pointing to its hemostatic potential. Overall, our results indicate that LG, likely through its capacity to modify membrane lateral organization, has important pro-aggregant and antihemorrhagic properties.

## Introduction

**Figure 1:**
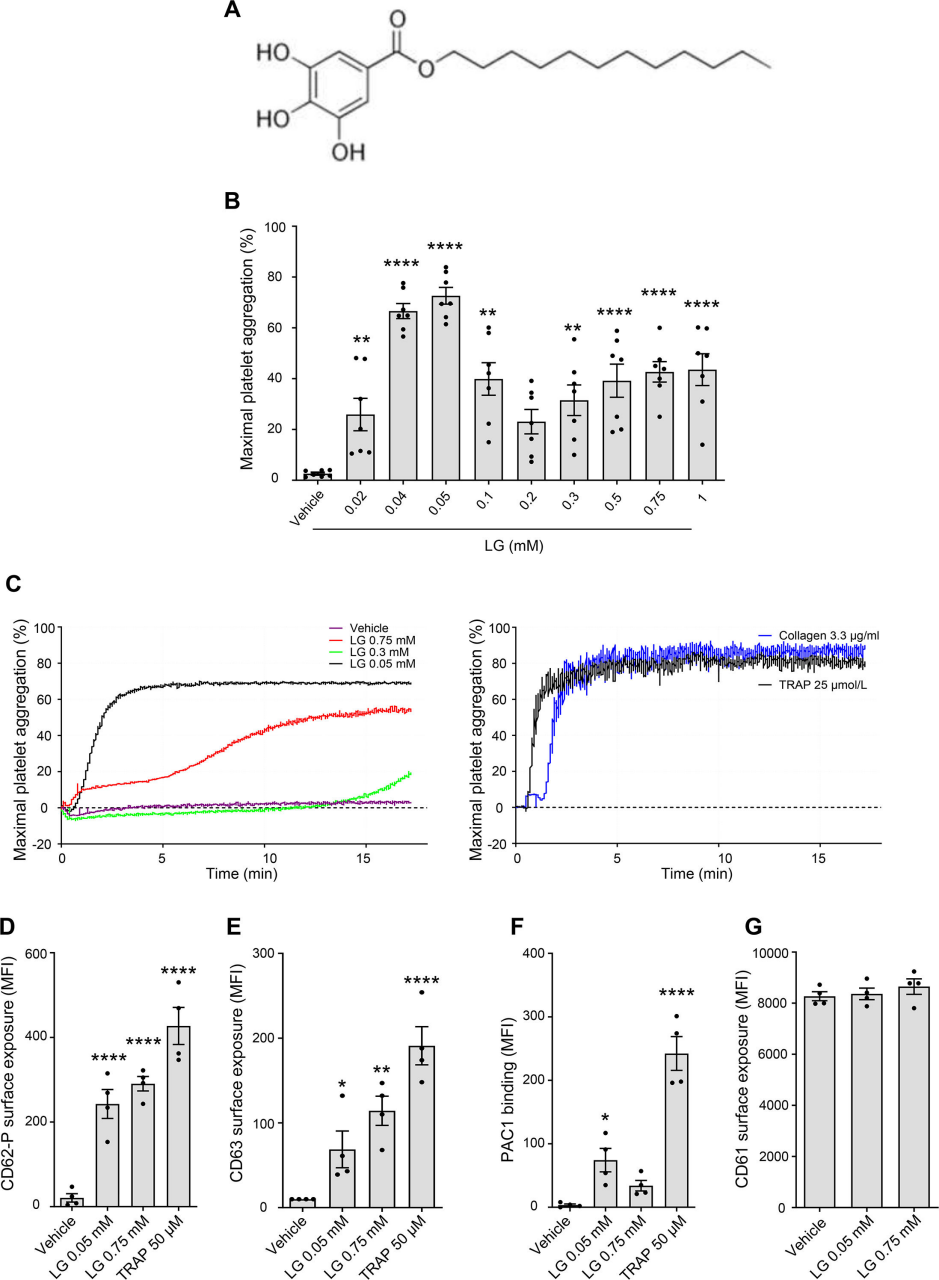
Effects of LG on washed platelet aggregation and secretion. (**A**) Chemical structure of lauryl gallate (LG). LG is the n-alkyl ester of gallic acid, an aromatic organic compound [1]. (**B**) Washed platelets from healthy donors were treated or not with LG at the indicated concentrations or vehicle (DMSO), and spontaneous aggregation was assessed by turbidimetry for 15 minutes. Results are expressed as a percentage of maximal platelet aggregation and are mean ± SEM of seven independent experiments (**P<0.01; ****P<0.0001 according to one-way ANOVA followed by Sidak’s multiple comparisons test). (**C**) Representative washed platelet aggregation curves induced by different concentrations of LG (left panel) or by collagen (3.3 µg/ml) and TRAP (25 µmol/l) (right panel) are shown. Platelet secretion of (**D**) α-granules (CD62-P) and (**E**) dense granules (CD63) was quantified by flow cytometry following LG or vehicle (DMSO) addition during 10 minutes. (**F**) Activation of the αIIbβ3 integrin (GpIIbIIIa) was measured using PAC-1 antibody by flow cytometry after 10 minutes of LG stimulation. (**G**) Total αIIbβ3 integrin surface expression was measured using CD61 (GpIIIa) antibody by flow cytometry after 10 minutes of LG stimulation. Results are expressed as median fluorescence intensity (MFI) and are mean ± SEM of four independent experiments (*P<0.05; **P<0.01; ****P<0.0001 according to one-way ANOVA followed by Sidak’s multiple comparisons test). TRAP, thrombin receptor activating peptide.

Lauryl gallate (LG) is the ester of dodecanol and gallic acid (Figure 1A) and is widely used as an antioxidant food additive under the code E312, and in cosmetics. LG, also known as dodecyl gallate, is a derivative of gallic acid, an aromatic organic compound that acts as a free-radical scavenger, preventing oxidative rancidity of food and the generation of superoxide radicals by inhibiting enzymatic peroxidation [1]. The relatively low toxicity of this molecule in normal cells has justified its use in the food industry as an antioxidant for more than fifty years [2]. LG is used as a food preservative also because of its antibacterial activity, specifically against Gram-positive bacteria like *Bacillus subtilis* or *Streptococcus mutans*, by inhibiting the membrane respiratory chain [3,4]. LG has sporicide properties as it can inactivate spores like those of *B. subtilis* which are challenging to control due to their intracellular location and resistance to ultraviolet light and heat. Finally, although LG does not have antifungal activity [1], it has a potent antiviral activity [5]. For these different reasons, LG is considered a valuable molecule in food preservation [6].

In addition, LG has been shown to exhibit anti-proliferative and pro-apoptotic activities in tumoral cell lines with selectivity for rapidly growing cells, where it disrupts the mitochondrial membrane potential, triggers caspase activation, and induces DNA degradation [7].

The screening of a chemical compound library to detect molecules with the potential to induce *in vitro* human blood platelet aggregation, performed in our laboratory, pointed to LG as a new potential proaggregating agent. So far, the effect of LG on hemostasis and thrombosis has remained unexplored. Here, we have focused our study on the impact of LG on blood platelets, which are critical players in hemostasis and thrombosis. Briefly, following vascular injury, platelets first interact with exposed sub-endothelial collagen via interaction with the von Willebrand factor (vWF) that binds to collagen fibers and the platelet GPIb-IX-V complex. Subsequently, platelet adhesion and activation are ensured through the triggering of two surface receptors for collagen, the integrin α2β1 and glycoprotein VI (GPVI) [8]. Platelets then undergo secretion of alpha and dense granules along with activation of the αIIbβ3 integrin. Activated platelets release adhesion proteins, coagulation factors, and soluble agonists including adenosine diphosphate, thromboxane A2, and serotonin that contribute to further amplify their signal transduction pathways and recruit new platelets [9]. Activation of αIIbβ3 by an inside-out signaling process allows binding of the integrin to circulating fibrinogen, which supports platelet–platelet interactions and, in turn, platelet aggregation. Platelet αIIbβ3 integrin outside-in signaling, co-ordinated with other platelet membrane receptor signals, ensures platelet thrombus formation, stability, and retraction, particularly in conditions of shear stress [10,11]. Platelets have several membrane receptors that can efficiently activate intracellular signal transduction pathways. The plasma membrane fluidity and lateral organization, including lipid raft dynamics, have very important impacts on platelet signaling [12]. Importantly, besides protecting against oxidative stress, LG, which possesses a C12 hydrocarbon chain, can be incorporated into biomembranes and influence the membrane rigidity and organization. Indeed, the fluidizing effect of LG described on artificial membranes may affect the lateral membrane organization, raft behavior and, in turn, signal transduction machinery [13].

Interestingly, we found that LG can efficiently induce platelet activation, secretion, and aggregation at low doses through the activation of various intracellular signaling pathways, including calcium signaling. Interestingly, the addition of a low dose of LG to human whole blood strongly promoted a platelet thrombotic response under arterial flow. At high concentrations, LG induces dramatic membrane modifications along with calcium influx and a slow process of platelet aggregation. Finally, a simple flash application of LG can efficiently decrease tail bleeding in rats, suggesting that this compound could be repositioned as a hemostatic and antihemorrhagic agent.

## Materials and methods

### Reagents

LG (IUPAC name dodecyl 3,4,5-trihydroxybenzoate, CAS number 1166–52-5) was purchased from Sigma Aldrich, certified as high purity [≥ 99.0% (HPLC)]. Thrombin receptor activating peptide 14 (TRAP), prostacyclin, and apyrase were also obtained from Sigma-Aldrich (Saint-Louis, U.S.A.). HORM collagen type I from equine tendon was from Takeda (St. Peter Strasse, Austria), 1,2-Bis(2-aminophenoxy)ethane-N*,*N*,*N*',*N*'*-tetraacetic acid tetrakis(acetoxymethyl ester) (BAPTA-AM) from Focus Biomolecule (Plymouth Meeting, U.S.A.), and calcifluor 8 AM from Santa Cruz Biotechnology (Dallas, U.S.A.). The Annexin V Alexa Fluor (AF) 647/PI cell apoptosis assay kit was from Clinisciences (Nanterre, France). For antibodies, anti-phospho-p38 MAP-kinase (ref#4511), anti-p38 MAP-kinase (ref#9212), anti-phospho-Akt (Ser 473) (ref#4060), anti-Akt (ref#9272), and anti-β-actin (ref#3700) were purchased from Cell Signaling Technology (Danvers, U.S.A.). PAC-1 FITC (Catalog No: 340507), anti-CD62P FITC (Catalog No: 550866), anti-CD63 FITC (Catalog No: 561924) and anti-CD61 PE (Catalog No: 555754) were obtained from BD Biosciences (Franklin Lakes, U.S.A.), and peroxidase-conjugated anti-mouse or anti-rabbit antibodies were from Promega (Madison, U.S.A.). All other chemicals and reagents used were of the highest commercially available purity. LG was suspended in dimethyl sulfoxide noted DMSO (LOBA Chemie, Ahmedabad, India) at 250 mM as the initial concentration. In all experiments, the percentage of DMSO did not exceed 0.25%.

### Blood sampling and platelet preparation

Blood samples were collected using Vacutainer tubes containing 3.2% sodium citrate from healthy volunteers who had abstained from anti-platelets or anti-inflammatory drugs for at least 10 days before blood sampling. Healthy volunteers [median age 31 (interquartile range, 22–54), 53.7% female] were recruited after informed consent under a protocol approved by the Toulouse Hospital Bio-Resources biobank, registered with the Ministry of Higher Education and Research (DC2016-2804). Blood was centrifuged for 10 minutes at 190 g, and the supernatant obtained was the platelet-rich plasma (PRP). Further centrifugation of the PRP at 3500 g for 10 minutes resulted in platelet-poor plasma in the supernatant.

Washed platelets were prepared from centrifuged PRP previously supplemented with prostacyclin (0.5 µM) to avoid platelet activation. The pellet was suspended in buffer A (pH 6.8) containing 140 mM NaCl, 5 mM KCl, 5 mM KH_2_PO_4_, 1 mM MgSO_4_, 10 mM N-2-hydroxyethylpiperazine-N’-2-ethanesulfonic acid, 5 mM glucose, and 0.35% bovine serum albumin (BSA) (w/v). The same buffer containing 1 mM CaCl_2_ and apyrase but without BSA (adjusted to pH 7.4) was added to the final suspension to reach 2 × 10^8^ platelets/ml.

### Platelet aggregation

Different concentrations of LG were added to 270 µl of washed platelets from an initial concentration of 250 mM. Platelet aggregation was recorded using a light transmission aggregometer (SD Medical, Stago, France). The percentage of transmittance of washed platelet suspension was recorded as 0%, and that of the appropriate buffer was 100%. Platelets treated with DMSO alone were considered as negative controls. Potentiation between low doses of LG and collagen or TRAP to induce platelet aggregation was also determined.

### Flow cytometry analysis of platelet-granule secretion and αIIbβ3 activation

Washed platelets were pre-incubated with LG or DMSO for 5 and 10 minutes at 37°C. Samples were then incubated for 15 minutes at room temperature (RT) in the dark with anti-CD62P (dilution 1/25), anti-CD63 (dilution 1/25), monoclonal PAC-1 antibody (dilution 1/25), which selectively recognizes the active form of the αIIbβ3 integrin, or anti-CD61 (dilution 1/25), which recognizes total αIIbβ3 integrin. Analysis of respective proteins was performed using a FACS-Verse cytometer (BD Biosciences, Franklin Lakes, U.S.A.). Platelets were identified based on their forward scatter and side scatter properties.

### Scanning electron microscopy

Platelets are treated with TRAP 25 µM and LG (LC and HC) for 10 minutes. They were fixed in 2.5% glutaraldehyde in 0.1 M Sorensen phosphate buffer (pH 7.4) for at least 1 hour at 37°C. After sedimentation, the pellets were resuspended in water and made to adhere onto poly-lysine-coated glass coverslips. Platelets were then dehydrated in a graded ethanol series and dried by critical point drying with a Leica EM CPD 300. The samples were coated with 6 nm platinum on a Leica EM Med 020 before being examined on a FEI Quanta 250 FEG scanning electron microscope at an accelerating voltage of 5 kV.

### Measurement of LDH leakage

Washed platelets from healthy donors were treated with different doses of LG or control (DMSO) for 10 minutes at 37°C. Following, platelets were pelleted by centrifugation at 1800 g for 3 minutes. Supernatants were used to detect lactate dehydrogenase (LDH) release by colorimetric method (ThermoFisher Scientific, Waltham, U.S.A.), according to the manufacturer’s protocol. The assay was performed in endpoint time of increased formazan absorbance at 680 nm for 30 minutes.

### Procoagulant platelet detection

Phosphatidylserine exposure was measured with annexin-V binding by flow cytometry using the appropriate kit (CliniSciences, Catalog No: FXP023-100) according to the manufacturer’s protocol. Briefly, washed platelets from healthy donors were treated with increasing concentrations of LG for 10 minutes at 37°C, and stimulated platelets (25 µl) were incubated with annexin-V AF647 for 30 minutes at 37°C. After adding the binding buffer, samples were analyzed using flow cytometry (50,000 events acquired).

### Western blotting

Washed platelets were treated with LG or DMSO, and aliquots were taken at 0, 2.5, 5, and 10 minutes and immediately lysed with 5× lysis buffer (225 mM Tris, pH 6.8, 50% glycerol, 5% sodium dodecyl sulfate, 0.25 M dithiothreitol, 5 mM EGTA, and 0.05% bromphenol blue). Platelet lysates were heated at 100°C for 3 minutes, submitted to sodium dodecyl sulfate 10% polyacrylamide gel electrophoresis (SDS-PAGE), and transferred onto nitrocellulose. The membrane was incubated in tris-buffered-saline containing 0.1% Tween (TBST) and 5% BSA (w/v) for 30 minutes before addition of either the rabbit antiphospho-Akt or antiphospho-p38 MAP-kinase (p38MAPK) specific antibodies (dilution 1/1000). After overnight incubation at 4°C, washing in TBST, and incubation with the horseradish peroxidase (HRP)-conjugated anti-rabbit secondary antibody (dilution 1/5000) in TBST containing 5% BSA (w/v) at RT for 1 hour, immunoblots were developed using Super-signal chemiluminescent substrate (Pierce, Rockford, U.S.A.). The membrane was then stripped using the glycine stripping buffer (1.5% w/v glycine, 1% SDS, 1% Tween-20 at pH 2.2 adjusted with HCl) for 30 minutes, washed once in PBS and twice in TBST for 10 minutes each and incubated in TBST containing 5% non-fat dry milk (w/v) for 30 minutes at RT. The mouse anti-β-actin antibody (dilution 1/3000) was then added together with the HRP-conjugated anti-mouse secondary antibody (dilution 1/5000) for another 30 minutes at RT. After washing, detection of β-actin, used as a normalizing signal, was performed using standard ECL reagent (Biorad). Quantitative phosphorylation analysis was performed by densitometric analysis with Chemidoc Imaging system (ImageLab, Biorad, Hercules, U.S.A.).

For the western blot showing all signals (phospho-Akt, phospho-p38MAPK, total Akt, total p38 MAP-kinase, and β-actin) from the same membrane, after SDS-PAGE, proteins were transferred onto PVDF membranes. After blocking in TBST containing 5% BSA for 30 minutes, the rabbit antiphospho-Akt or antiphospho-p38MAPK-specific antibodies (dilution 1/1000) were added and incubated overnight at 4°C. After three washes in TBST for 10 minutes each, the membrane was incubated in TBST containing 5% BSA (w/v) for 30 minutes. The HRP-conjugated anti-rabbit secondary antibody (dilution 1/5000) was added and immunoblots were developed using ECL Max detection reagent. The membrane was then carefully cut horizontally to separate the regions containing Akt and p38MAPK signals and stripped with the glycine stripping buffer (1 hour for Akt and 30 minutes for p38MAPK). After washing in PBS and TBST and blocking in TBST containing 5% non-fat dry milk (w/v) for 30 minutes, rabbit anti-Akt and anti-p38MAPK specific antibodies were added (dilution 1/1000) and incubated overnight at 4°C. The membranes were then washed three times for 10 minutes in TBST and incubated in TBST containing 5% non-fat dry milk (w/v) for 30 minutes. HRP-conjugated anti-rabbit secondary antibody (dilution 1/5000) was added for 1 hour at RT, and after washing, immunoblots were developed using ECL Max detection reagent. Finally, the membrane containing the p38MAPK signal was stripped once more with the glycine stripping buffer for 1 hour, washed with PBS and TBST, and reprobed with the specific mouse anti-β-actin antibody (dilution 1/3000) incubated together with the HRP-conjugated anti-mouse secondary antibody (dilution 1/5000) in TBST containing 5% BSA (w/v) for 30 minutes at RT. After washing, detection was performed using ECL detection reagent.

### cAMP assay

Quantification of intracellular cyclic adenosine monophosphate (cAMP) was performed using the homogeneous time-resolved fluorescence (HTRF) cAMP competitive immunoassay (cAMP-Gi kit, Revvity, France) according to the manufacturer’s instructions. Briefly, 150,000 washed platelets were distributed in a 384-well white microplate (Greiner Bio-One, Les Ulis, France) and incubated or not (vehicle) with forskolin alone (Fsk) or in combination with LG (0.05 mM) or epinephrine (10 µM) for 45 minutes at 37°C and in the presence of 0.5 mM IBMX to prevent phosphodiesterase-mediated cAMP degradation. After the addition of cryptate-labeled cAMP (donor) and the anti-cAMP-d2 (acceptor) for 1 hour, the specific FRET signals were calculated by the fluorescence ratio of the acceptor and donor emission signal (665/620 nm) collected using a modified Infinite F500 (Tecan Group Ltd, Mannedorf, Switzerland). Conversion of the HTRF ratio of each sample into cAMP concentrations was performed based on a standard curve to determine the linear dynamic range of the assay.

### IP1 assay

Quantification of intracellular inositol 1 phosphate (IP1) was performed using the HTRF IP1 competitive immunoassay (IP-One Tb kit, Revvity, France) according to the manufacturer’s instructions. Briefly, 300,000 washed platelets were distributed in a 384-well white microplate (Greiner) and incubated or not (vehicle) with LG (0.05 mM) or TRAP (50 µM) for 2 hours at 37°C in the presence of 50 mM of LiCl to prevent IP1 degradation. After the addition of d2-labeled IP1 (acceptor) and anti-IP1-cryptate (donor) for 1 hour, the specific FRET signals were calculated by the fluorescence ratio of the acceptor and donor emission signal (665/620 nm) collected using a modified Infinite F500 (Tecan Group Ltd, Mannedorf, Switzerland). Conversion of the HTRF ratio of each sample into IP1 concentrations was performed based on a standard curve to determine the linear dynamic range of the assay.

### Calcium mobilization

Platelet calcium flux was adapted from the work described by Monteiro et al. [14]. Washed platelets resuspended at 10^8^/ml in platelet wash buffer were incubated with the calcium indicator dyes Calcifluor 8-AM (R&D system) at 5 µM and Pluronic F-127 0.02% v/v (Sigma Aldrich, Saint Louis, U.S.A.) for 30 minutes at 37°C. The platelets were washed twice with platelet wash buffer and resuspended in Tyrode’s buffer containing 1 mM of ethylene glycol-bis(β-aminoethyl ether)-*N*,*N*,*N*′,*N*′-tetraacetic acid (EGTA) or BAPTA-AM (10 μM). Changes in cytosolic calcium concentration were associated with increased or decreased fluorescence emission of Calcifluor 8-AM (emission wavelengths of 500–570 nm). These changes were analyzed using flow cytometry BD LSRII Fortessa. Results were expressed in fold-increased median fluorescence. The determination of internal calcium concentration in platelets was described by Grynkiewicz et al. [15].

### Thrombus formation on immobilized collagen under flow conditions

This method was performed as previously described [16]. Briefly, biochips with micro-capillaries (Vena8Fluoro+®, Cellix, Dublin, Ireland) were coated with collagen type I. An epi-fluorescence microscope (Axio Observer; Carl Zeiss, Jena, Germany) allowed direct visualization of platelet adhesion and aggregation, which was recorded with an ORCA-R2 camera (Hamamatsu, Japan). Whole blood was collected into 13.6 UI/ml lithium heparinate, and platelets were labeled with 2 μM DIOC6 (Life Technologies, Carlsbad, U.S.A., 10 minutes at 37°C). LG (0.05 mM) was then added to blood just before injection through a micro-capillary at a shear rate of 1500 s^−1^. Thrombus formation was visualized with a 40× long working distance objective in real-time. The range was fixed at 80 µm and the interval at 1.5 µm. The acquisition rate was chosen at one frame every 30 seconds and divided into two positions. Quantification of surface coverage and thrombi volume was performed offline using ImageJ software.

### Rat tail bleeding time

Wistar rats of either gender weighing between 200 and 250 g were purchased from the Central Pharmacy of Tunisia. Experimental protocols were carried out at the animal facility of the Center of Biotechnology of Sfax in compliance with the approval of the animal experiments, which was obtained from the Medical Ethics Committee for the Care and Use of Laboratory Animals of the Pasteur Institute of Tunis, Tunisia (approval number: FST/LNFP/Pro 152012). Animals were fed a standard chow diet and had free access to water. They housed five rats in a cage (0.12 m^2^). Phenobarbital was used to anesthetize rats at a dosage of 25 mg/kg through intraperitoneal injection [17]. Bleeding time was measured by transection of the tail, 1 cm from the tip. Immediately after transection, the tail was dipped for 5 seconds into LG solution (5, 50, or 100 mg/ml of LG dissolved in ethanol) or vehicle (ethanol) and then placed carefully on filter paper. Blood was allowed to drop on filter paper, which was moved gently every 30 seconds until no more blood was dropped. If bleeding did not resume within 30 seconds of cessation, it was considered stopped [16]. The bleeding time was also determined for rats not treated with any solution (control) and for rats treated with ethanol to test the effect of the solvent. Tranexamic acid (100 mg/ml) was used as a standard anti-hemorrhagic drug under the same conditions. Each condition was repeated for six rats (44% females). We mention that animals were not euthanized; we kept them until their tails healed, and then they were used for breeding.

### Statistical analysis

Results were presented as the mean ± SEM of at least three independent experiments. Statistical significance among groups was analyzed by the two-way ANOVA test, Mann-Whitney test, or Kruskal-Wallis test using Graph Pad Prism program [*P*<0.05 (*); *P*<0.01 (**); *P*<0.001 (***); *P*<0.0001 (****)].

## Results

### LG induces platelet aggregation and granule secretion

LG, the ester of dodecanol and gallic acid (Figure 1A), was first tested alone at increasing concentrations on washed human platelet aggregation response. As shown in Figure 1B, LG induced a non-monotonic dose response with a first wave of platelet aggregation response at low concentrations (with a peak reaching nearly 80% of maximal aggregation at 0.05 mM) followed by a decrease in platelet aggregation at higher concentrations (until 0.2 mM). Beyond this point, there was again an increase in platelet aggregation response at high doses, reaching a plateau between 0.75 and 1 mM. Platelet aggregation induced by 0.05 mM LG was comparable to that classically observed following stimulation by physiological platelet agonists such as TRAP and collagen, reaching a maximal aggregation response within 3 minutes and remaining stable (Figure 1C). In contrast, a slow platelet aggregation response, hardly reaching 50% of maximal aggregation, was observed when high concentrations of LG were used (Figure 1C). We then investigated the surface expression of the platelet α-granule secretion marker CD62-P (P-selectin) and the dense granule secretion marker CD63 by flow cytometry. The surface expression of CD62-P on washed platelets was markedly increased following the addition of LG at 0.05 mM or 0.75 mM (Figure 1D). For the sake of comparison, the intensity of α-granule secretion induced by LG was about 25% lower than that induced by 50 µM of thrombin receptor agonist peptide (TRAP). Although less efficient than TRAP, LG also significantly increased CD63 surface expression level (Figure 1E). These results indicate that LG can activate platelets and induce the secretion of their alpha and dense granules. Moreover, washed platelets stimulated by LG showed a significant increase in the expression of the active form of the fibrinogen receptor αIIbβ3, particularly at the low dose of 0.05 mM, without modification of total αIIbβ3 surface expression (Figure 1F and G). The activation of αIIbβ3 by LG at 0.05 mM was, however, 50% lower than that induced by 50 µM TRAP.

**Figure 2:**
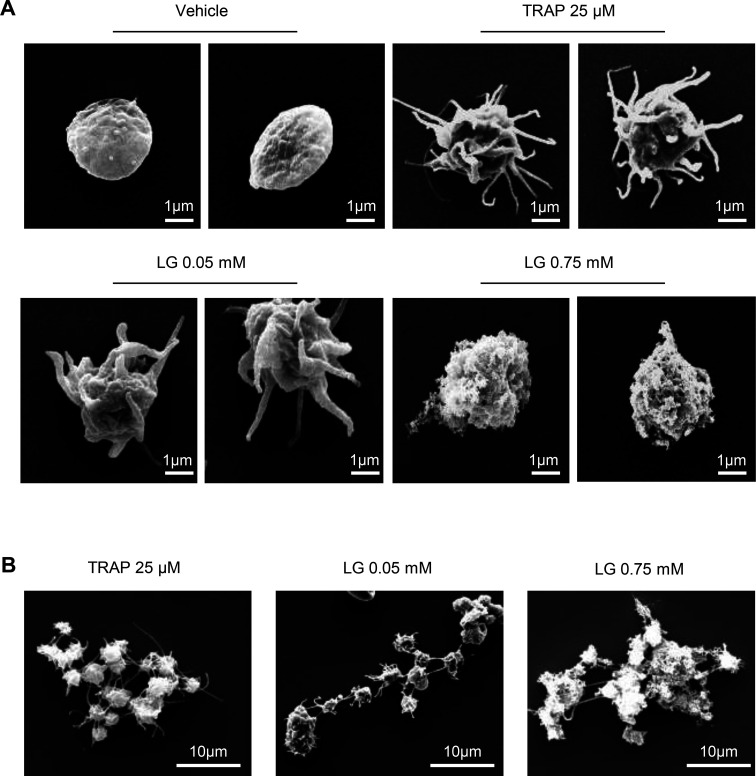
Scanning electron microscopy of platelets following stimulation with LG or TRAP. (**A**) Representative scanning electron micrographs of washed platelets from healthy donors treated with DMSO (vehicle), TRAP 25 µM, and LG 0.05 mM and 0.75 mM during 10 minutes at 37°C in non-shaking conditions. (**B**) Platelets were stimulated in similar conditions but under shaking conditions to allow platelet aggregation. Images of individual platelets (**A**) and platelet aggregates (**B**) are representative of three different healthy donors.

Taken together, these data demonstrate that LG induced spontaneous platelet activation, secretion, and aggregation. However, the profile of aggregation response was different when low (0.05 mM) or high (0.75 mM) doses of LG were used, suggesting a different mechanism of platelet activation. Knowing that LG could disturb platelet membrane organization, we analyzed the morphology of washed platelets treated with LG at 0.05 mM or at 0.75 mM by scanning electron microscopy. As expected, resting platelets from healthy donors were discoid, while TRAP-stimulated platelets, in non-shaking conditions, changed their shape and emitted filopodia (Figure 2A). Interestingly, LG at 0.05 mM also induced platelet shape change and filopodia formation, although the platelet body was less contracted and filopodia were thicker compared with TRAP-stimulated platelets. At a high concentration of LG (0.75 mM), platelets also changed their shape, but filopodia were no longer visible, and the membrane structure was strongly modified with a grainy appearance and apparent pore formation (Figure 2A). Under shaking conditions, platelet aggregates were formed in all cases with again different morphology of platelets (Figure 2B). Platelet aggregates induced by LG at 0.05 mM closely resembled those induced by TRAP, while platelet aggregates induced by LG at 0.75 mM exhibited distinct characteristics, with fewer filopodia and a grainy appearance.

The potential damage to the cell membrane was further investigated by measuring LDH leakage following treatment of washed platelets with increasing doses of LG (Supplementary Figure S1). At low doses of LG, no significant LDH release from platelets was detected, consistent with the intact membrane structure observed by electron microscopy. However, at higher doses of LG (>0.5 mM), a significant proportion of LDH (ranging from 10 to 20% of the total LDH) was detected in the supernatant, confirming that LG caused platelet membrane damage and porosity, as observed in the scanning electron micrographs. We then investigated whether LG would induce procoagulant platelet formation by measuring annexin V binding to phosphatidylserine (PS) exposed on the outer leaflet of the platelet plasma membrane (Supplementary Figure S1). At 0.05 mM, LG did not induce a significant exposure of PS on the platelet surface, indicating that it was not able to promote platelet procoagulant activity. Of note, a strong signal was observed at 0.75 mM LG possibly due to the permeability of the membrane allowing annexin V-FITC to enter platelets.

### LG initiates signal transduction pathways in human platelets

**Figure 3:**
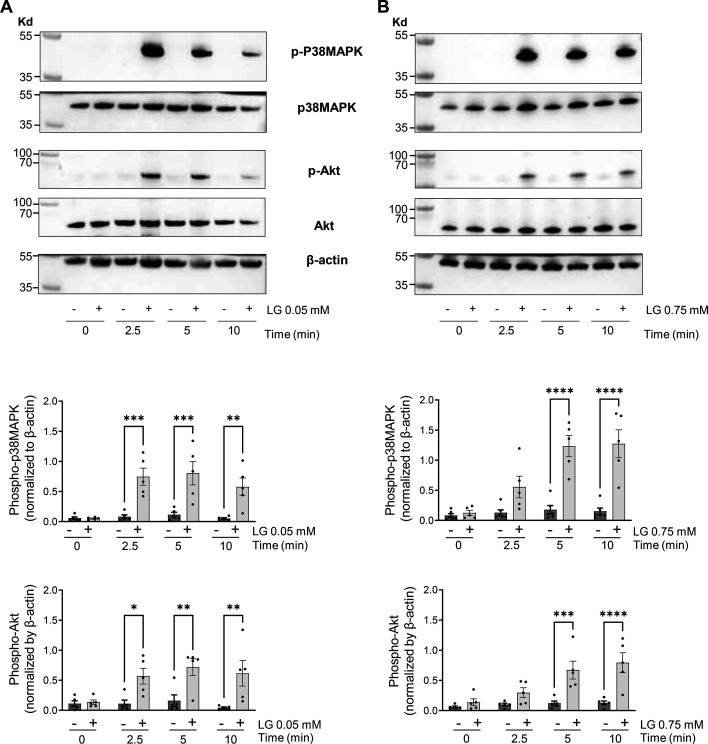
Effects of LG on signal transduction events. The effect of LG at 0.05 mM (**A**) and 0.75 mM (**B**) on washed human platelet Akt and p38MAPK activation was analyzed by Western blotting using specific anti-phospho-antibodies (Akt phosphorylation on Ser-473, p38MAPK phosphorylation on Thr180/Tyr182) and antibodies against total Akt, total p38MAPK, and total β-actin. Images showing phospho-Akt, phospho-p38MAPK, total Akt, total p38MAPK, and β-actin signals on the same Western blot (upper panel) are from one donor representative of three. Samples were processed as detailed in the Materials and Methods section. For quantification, performed by densitometric analysis (lower panel), anti-phospho-Akt and anti-phospho-p38MAPK antibodies were stripped, and the membranes were reprobed with the anti-β-actin antibody, used as a normalization signal. Results are mean ± SEM of five independent experiments (*P<0.05; **P<0.01; ***P<0.001; ****P<0.0001 according to two-way ANOVA followed by Sidak’s multiple comparisons test).

**Figure 4:**
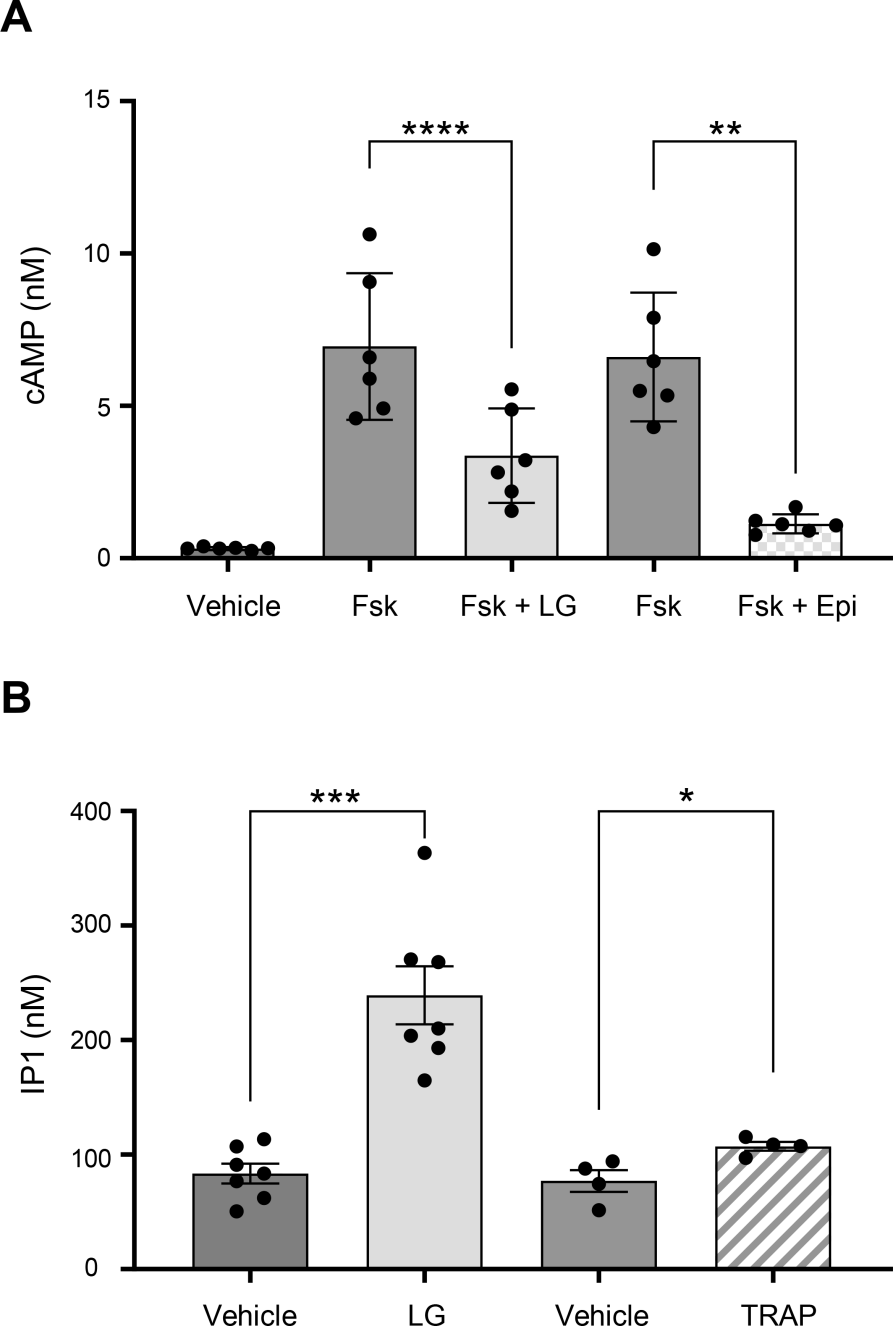
LG induces cAMP decrease and inositol phosphate increase. (**A**) Washed human platelets were stimulated or not (vehicle) with 5 µM forskolin (Fsk) alone or in the presence of 0.05 mM LG (Fsk + LG) or 10 µM epinephrine (Fsk + Epi) for 45 minutes, and the intracellular cAMP level was quantified. Data represent the mean ± SEM of 6 healthy donors and are expressed as cAMP concentration (nM). The statistical comparison was assessed using one-way ANOVA followed by Sidak’s multiple comparison test (**P<0.01; ***P<0.001). (**B**) Washed human platelets were stimulated or not (vehicle) with 0.05 mM LG or 50 µM TRAP for 2 hours, and intracellular inositol 1 phosphate (IP1), a degradation product of IP3, was quantified. Data represent the mean ± SEM of 4 to 7 healthy donors and are expressed as IP1 concentration (nM). The statistical comparison was assessed using one-way ANOVA followed by Sidak’s multiple comparison test (*P<0.05; ***P<0.001).

**Figure 5:**
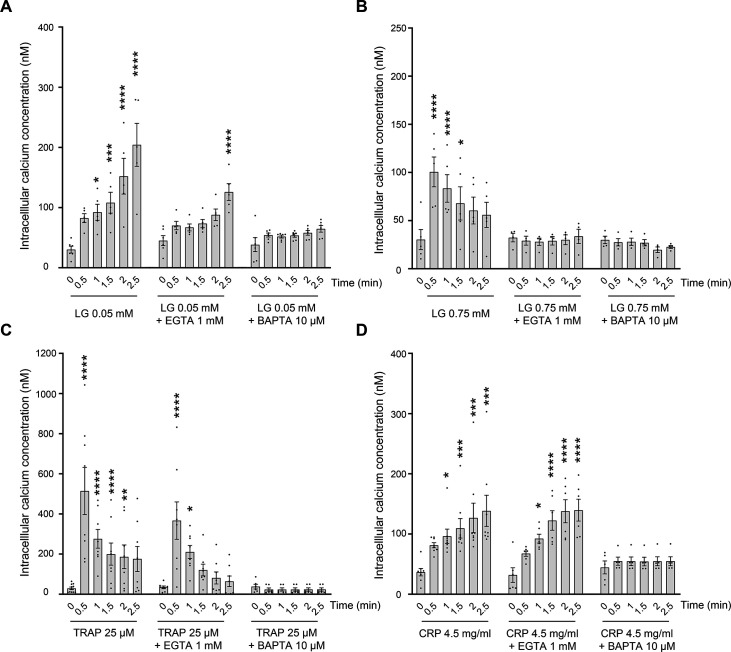
Effect of LG on cytosolic calcium concentration. Washed platelets pretreated with CalciFluor 8-AM were stimulated by LG at 0.05 mM (**A**) or 0.75 mM (**B**), TRAP 25 µM (**C**) or CRP 4.5 µg/ml (**D**) in the presence or not of BAPTA-AM 10 µM, EGTA 1 mM or vehicle (DMSO). Free cytosolic calcium concentration was monitored by flow cytometry for 3 minutes. Results are expressed as cytosolic calcium concentration (nM) and are mean ± SEM of four to seven independent experiments (*P<0.05; **P<0.01; ***P<0.001; ****P<0.0001 according to one-way ANOVA followed by Sidak’s multiple comparison test).

Having shown that platelets form aggregates following LG addition, we next investigated the potential activation of intracellular signal transduction pathways. We first analyzed the phosphorylation of Akt at Ser473 and p38MAPK at Thr180/Tyr182 by Western blotting. Consistent with the rapid induction of platelet aggregation, 0.05 mM of LG induced a rapid activation of Akt and p38MAPK phosphorylation (Figure 3A). Conversely, the high dose of LG (0.75 mM) induced Akt and p38MAPK activation at relatively late times (5 and 10 minutes), corroborating the slower platelet aggregation response observed in this condition (Figure 3B). These data indicate that LG can trigger intracellular platelet signaling pathways, with differences in velocity at low and high concentrations. To further characterize LG-induced intracellular signaling pathways, we investigated the cAMP and inositol phosphate pathways. Interestingly, LG at 0.05 mM was almost as efficient as 10 µM epinephrine at reducing forskolin-induced cAMP rise through inhibition of adenylate cyclase, suggesting an activation of the heterotrimeric Gi protein (Figure 4A). Furthermore, IP1, a metabolite of IP3, accumulated in platelets treated by LG at 0.05 mM, indicating activation of the phospholipase C pathway (Figure 4B). For comparison, LG was more efficient than TRAP at inducing IP1 production. Therefore, we then investigated calcium signaling using CalciFluor (Fluo-8) to label free cytosolic calcium and flow cytometry detection. We compared the effect of LG on washed human platelets to that induced by 25 µM TRAP or 4.5 µg/ml collagen-related peptide (CRP). As illustrated in Figure 5A, LG at 0.05 mM induced a robust increase in cytoplasmic free calcium concentration (reaching 230 **±** 25 nM after 2.5 minutes) comparable in kinetics and intensity to that induced by CRP (Figure 5D). This increase in cytosolic free calcium concentration was blocked by the intracellular calcium chelator BAPTA-AM and partially inhibited by the addition of EGTA, indicating that the low dose of LG induced both a calcium mobilization and calcium influx. The addition of LG at 0.75 mM induced a rapid increase in free cytosolic calcium, peaking at 105 **±** 15 nM within 0.5 minutes, followed by a decrease over time (Figure 5B). This rapid cytosolic calcium increase was abrogated by both BAPTA-AM and EGTA. However, since EGTA could have entered platelets due to pore formation in the plasma membrane at 0.75 mM LG, it is difficult to settle between an influx and/or a mobilization of calcium. As expected, TRAP induced rapid and transient calcium responses attributable to both calcium mobilization and influx (Figure 5C), while CRP initiated a slower but sustained calcium response largely due to calcium mobilization (Figure 5D).

### LG significantly enhances platelet aggregation and thrombus formation in whole blood under flow

**Figure 6:**
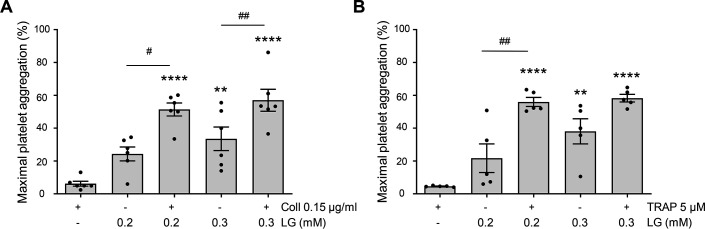
LG potentiates platelet aggregation induced by low doses of collagen or TRAP. Washed platelets were treated with collagen 0.15 µg/ml (**A**) or TRAP 5 µM (**B**) in the presence or not of LG at 0.2 or 0.3 mM and platelet aggregation was assessed by turbidimetry for 10 minutes. These concentrations of LG were chosen because they induced weak platelet aggregation (see Figure 1B). Results, expressed as percentage of maximal aggregation, are mean ± SEM of six (**A**) and five (**B**) independent experiments (vehicle versus LG with or without low dose of agonist **P<0.01; ****P<0.0001; LG alone versus LG with a low dose agonist ^#^ *P*<0.05, ^##^ *P*<0.01 according to one-way ANOVA followed by Sidak’s multiple comparisons test).

We then checked whether LG would potentiate platelet aggregation induced by low doses of collagen (0.15 µg/ml) or TRAP (5 µM). Two doses of LG (0.2 and 0.3 mM) that were poorly efficient in triggering washed platelet aggregation (Figure 1B) were used. While 0.15 µg/ml of collagen and 5 µM of TRAP alone induced a faint platelet aggregation response, the association with LG induced a significant potentiation of the response reaching 50 to 60% of the maximal platelet aggregation (Figure 6A and B).

**Figure 7:**
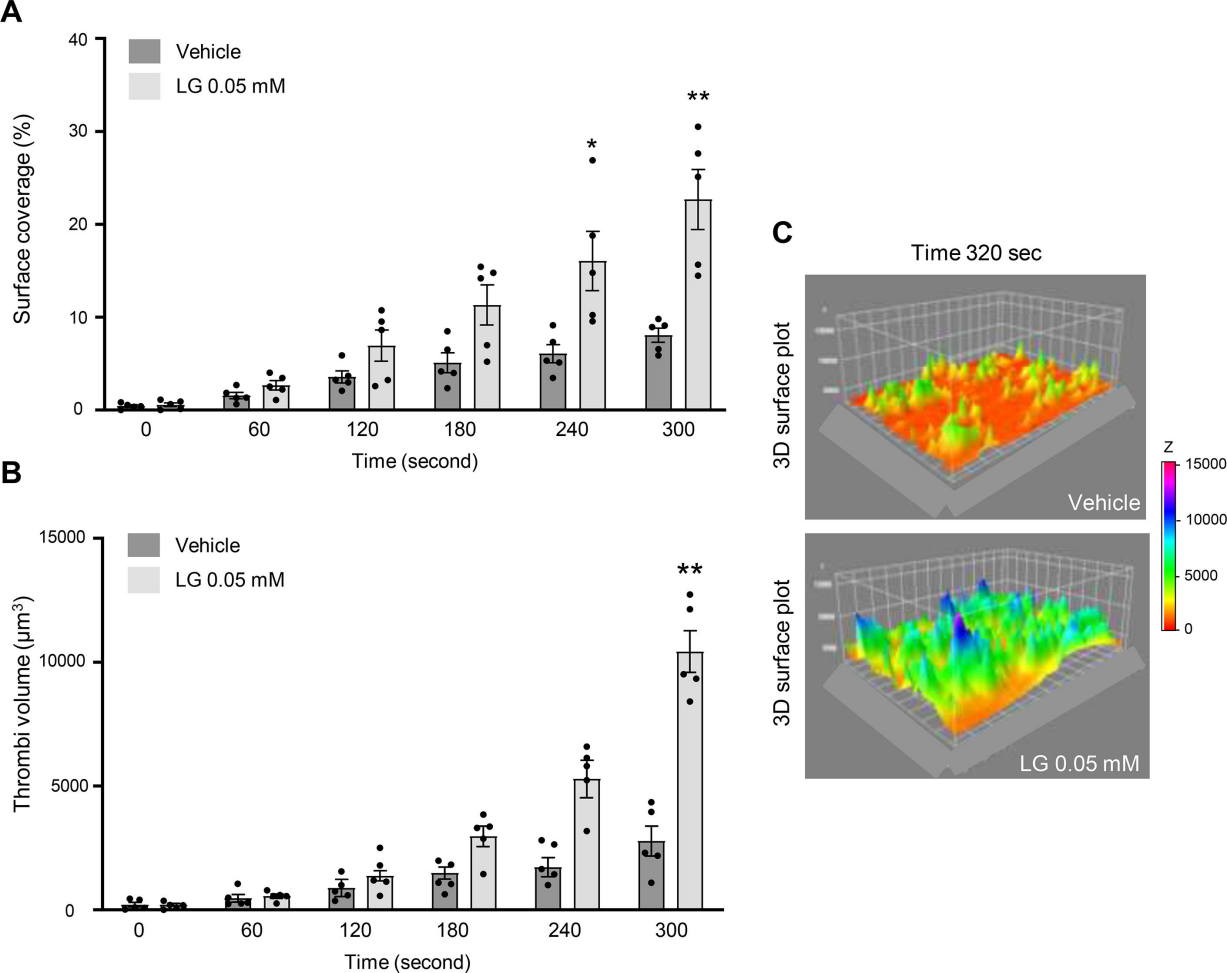
LG significantly potentiates thrombus formation in whole blood at arterial shear rate. DIOC_6_-labeled platelets in whole blood from healthy donors in the presence of LG 0.05 mM or vehicle (DMSO) were perfused through a collagen-coated microcapillary at a physiological arterial shear rate of 1500 s^−1^. Surface coverage (**A**) and thrombus volume (µm^3^) (**B**) were analyzed using ImageJ software. Graphs represent the averages of five independent experiments and are mean ± SEM (*P<0.05, **P<0.01 according to two-way ANOVA followed by Sidak’s multiple comparisons test). A representative 3D image of the thrombi formed after 320 seconds of blood flow is shown (**C**). The stability of LG (0.05 mM, 37°C during 15 minutes) in human blood was analyzed using gas chromatography coupled to mass spectrometry (GC-MS) and appropriate standards. The results indicate that 89.5% of LG was not metabolized under these conditions, while 10.5% was transformed into dodecyl alcohol.

To further investigate the effect of LG at a low dose (0.05 mM) on hemostasis, we performed a microfluidic-based thrombus formation assay using human whole blood perfused at physiological arterial shear stress on collagen-coated microcapillaries. Platelets were labeled with DIOC6, and the accumulation of fluorescent platelets on the collagen-coated surface was recorded by a video microscopy system, allowing the quantification of the surface covered by platelets and the thrombus volume. The addition of 0.05 mM LG to the blood significantly increased both the surface coverage (Figure 7A) and the thrombus volumes (Figure 7B) compared to control. The 3D surface plot shows the potent effect of 0.05 mM LG on platelet thrombus formation and growth (Figure 7C). Of note, in several experiments, the effect of LG on thrombus formation was so strong that the capillaries became occluded before the end of the recording (300 s).

### LG application strongly reduces the tail bleeding time in rats

**Figure 8:**
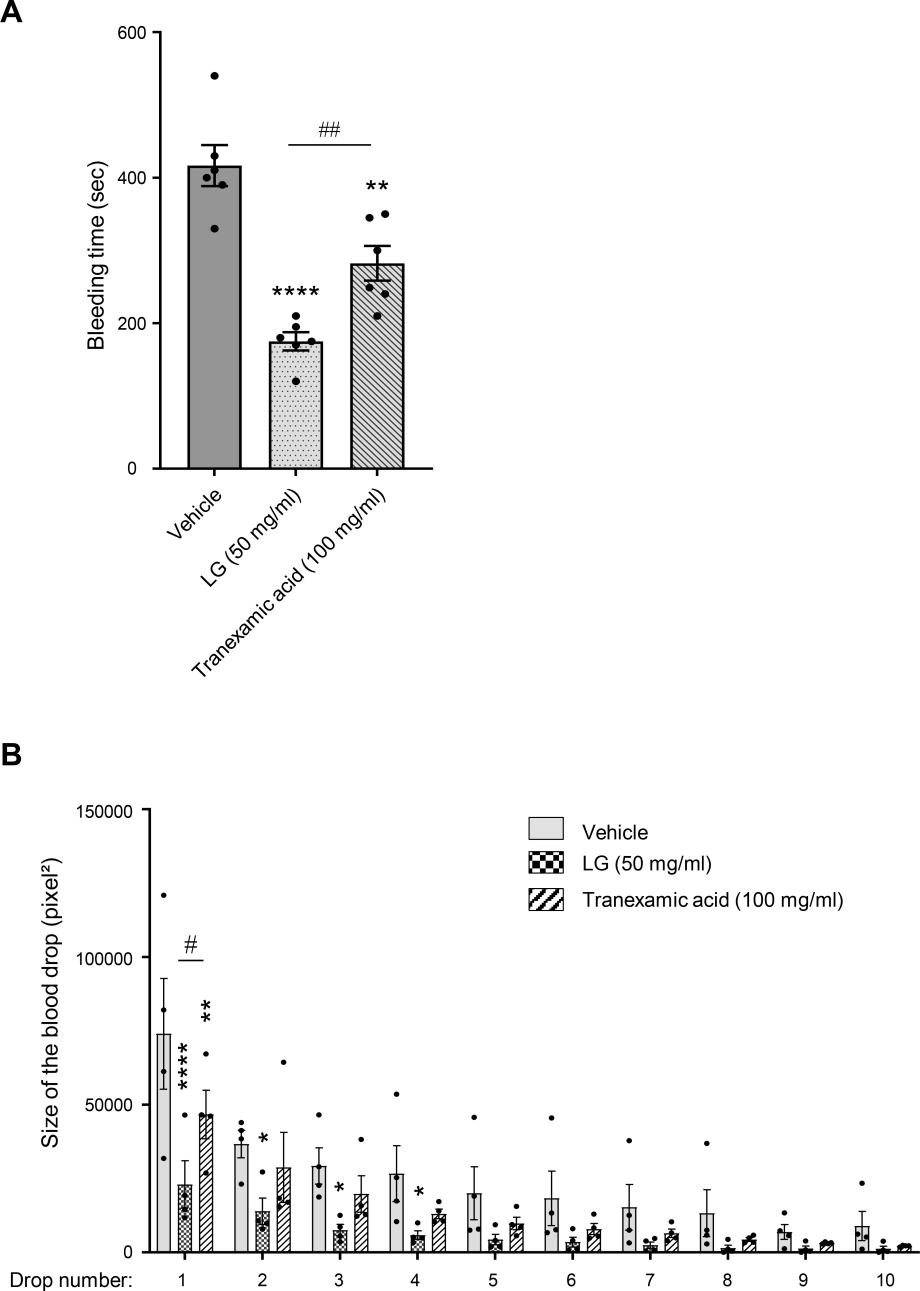
Application of LG strongly decreases the rat-tail bleeding time. Immediately after the transection cut, the rat tail was dipped for 5 seconds into ethanol (vehicle) or ethanol containing 50 mg/ml of LG. (**A**) The bleeding time was then indirectly determined using a filter paper to assess blood oozing from the wound without disturbing the forming clot. Tranexamic acid, a known antihemorrhagic solution, was used at a standard concentration of 100 mg/ml in ethanol. Results are expressed in seconds for the bleeding times and are mean ± SEM of six independent experiments. (**B**) Areas of the first ten drops collected on the filter paper (pixel²) are also provided, and results are mean ± SEM of four independent experiments (vehicle versus LG or tranexamic acid *P<0.05; **P<0.01; ****P<0.0001; LG versus tranexamic acid ^#^ *P*<0.05; ^##^ *P*<0.01 according to one-way ANOVA followed by Sidak’s multiple comparisons test). Of note, no influence of sex (44% female, 56% male) on the results of the study could be observed.

To assess the hemostatic properties of LG *in vivo*, we conducted a tail-bleeding assay in rats. After the section, the tail was dipped in an LG solution for 5 seconds, and the bleeding time was evaluated by gently dabbing with a Whatman paper until the bleeding stopped. Tranexamic acid, an antifibrinolytic compound well-known to reduce bleeding, was used for the sake of comparison. Interestingly, the LG application dramatically reduced the bleeding time, surpassing the efficacy of tranexamic acid in this murine bleeding model (Figure 8A). As shown in Figure 8B, the action of LG was also very fast.

## Discussion

LG is widely used in the food industry as an antioxidant, particularly for preventing the oxidative rancidity of foods containing fats and oils [1]. Following the discovery of its antibacterial and antiviral actions, numerous other functions have been reported, including anti-proliferative and anti-tumor activities [7,18]. In this study, we demonstrate that LG can induce human platelet shape change and filopodia formation, granule secretion, and platelet aggregation. At a concentration of 0.05 mM, LG induced a rapid platelet aggregation, which remained stable and comparable to that induced by physiological agonists. LG-induced platelet activation was associated with the secretion of both α and dense granules and with the activation of the αIIbβ3 integrin, an essential step allowing platelet aggregation through fibrinogen binding. These platelet responses were associated with a significant activation of intracellular signal transduction pathways, including phosphorylation of Akt, a well-known effector of class 1 phosphoinositide 3-kinase [19], p38MAPK activation [20], PLC activation, and calcium signaling [21], as well as inhibition of adenylate cyclase [22]. At a low dose, LG induced a rapid rise in cytosolic calcium, resulting from both calcium mobilization and calcium influx. This calcium response exhibited similar kinetics and intensity to that induced by the GPVI agonist CRP, which is, however, mainly due to calcium mobilization. Intracellular free calcium elevation is central to platelet activation, as it controls many molecular mechanisms, including phospholipase A2 activation, scramblase activity, or cytoskeleton organization, and in turn key responses such as granule secretion, platelet aggregation, and procoagulant activity [21,23].

However, it is important to note that, at a high concentration (0.75 mM), LG has a different effect on platelets, as it induces a dramatic plasma membrane modification. Indeed, scanning electron microscopy revealed a grainy appearance and pore formation, as also suggested by LDH measurement. These effects are in line with the hydrophobic nature of LG, which has been shown to insert into biological membranes and to alter their organization [13]. Platelet aggregation induced by the high concentration of LG occurred slowly, with a maximal aggregation barely reaching 50%. Contrary to the low dose of LG, in most cases, platelets did not extend filopodia, and the rapid rise in cytosolic calcium was likely primarily due to calcium influx. At the low dose, LG was able to trigger platelet activation without detectable membrane modification by electron microscopy or LDH release. Platelet activation induced by the low dose of LG was comparable to that induced by physiological agonists, suggesting that it could activate platelet receptors, either through direct interaction or, more likely, by altering the fluidity of the plasma membrane, leading to receptor clustering and activation, independently of ligands. Changes in membrane fluidity modify the capacity of lipids and proteins to diffuse laterally in the plane of the membrane and may induce lipid raft coalescence and clustering of membrane receptors [24]. The non-monotonic dose response of LG on platelet aggregation response and our electron microscopy data are consistent with such effect of LG on the platelet plasma membrane. Moreover, close to LG, propyl gallate, an ester formed by the condensation of gallic acid and propanol, also used in food preservation, has been shown to induce platelet aggregation [25], possibly by modifying the platelet membrane [26].

Interestingly, LG was not only active on washed human platelets, since the addition of 0.05 mM LG to human whole blood significantly enhanced the thrombotic response of platelets on a collagen matrix under arterial shear rate. Both the surface covered by platelets and the volume of platelet thrombi formed on the collagen matrix within the microfluidic system were significantly increased in the presence of LG. This result is consistent with the observation that LG potentiated the responses of washed platelets induced by subthreshold doses of collagen or TRAP, suggesting synergy of action. These results led us to assess the impact of a flash LG application on hemostasis in a rat-tail bleeding model. In line with the results obtained in vitro within the microfluidic system, the application of LG after the tail section significantly reduced the bleeding time. The concentration of LG used for these *in vivo* local application experiments was higher than that used *in vitro,* as plasma proteins and tail tissues, rich in extracellular matrices, are expected to induce important absorption, limiting the fraction of active compound reaching the target site [27]. In the rat-tail bleeding model, LG action was rapid and significantly more effective than the antifibrinolytic agent tranexamic acid. These data suggest that LG possesses hemostatic and antihemorrhagic potential when applied to a wound. Although the concentration of LG that may be present in the blood after ingestion of food containing this antioxidant is not documented and may be very low [28], one cannot exclude that excessive intake of this food additive could potentially increase the risk of thrombosis.

## Conclusion

This study represents the first documentation of the potent effect of LG on washed human platelet activation and platelet thrombus formation in whole blood under arterial shear rate. LG activates platelet intracellular signaling, particularly calcium signaling, possibly through modifications of membrane lateral organization. Its hemostatic and antihemorrhagic properties confirmed in vivo suggest a potential repositioning of this molecule for local therapeutic applications to prevent blood loss.

**Clinical Perspectives**

- There is still a need to develop an effective, available, and low-cost method to stop bleeding, easy to handle by a non-medical body in an emergency or medical setting.
- Lauryl gallate (LG) was found to strongly promote platelet activation and thrombus formation, significantly reducing bleeding time in rats by up to 50% and outperforming the antifibrinolytic agent tranexamic acid.
- These findings point to pro-aggregant and antihemorrhagic properties of LG and suggest its therapeutic application as a promising hemostatic product.

## Supplementary material

online supplementary figure 1.

Uncited online supplementary figure 2.

## Data Availability

All supporting data are included within the main article and its supplementary files.

## Open Access

This article has been published open access under our Subscribe to Open programme, made possible through the support of our subscribing institutions, learn more here: https://portlandpress.com/pages/open_access_options_and_prices#conditional

## Abbreviations

BSA: bovine serum albumin
CRP: collagen-related peptide
Fsk: Forskolin
GPVI: glycoprotein VI
HRP: horseradish peroxidase
HTRF: homogeneous time-resolved fluorescence
IP1: inositol 1 phosphate
LDH: lactate dehydrogenase
LG: lauryl gallate
PRP: platelet-rich plasma
PS: phosphatidylserine
RT: room temperature
TRAP: thrombin receptor agonist peptide
cAMP: cyclic adenosine monophosphate
cAMP: cyclic adenosine monophosphate
