# Supplementary material for: Lauryl gallate promotes platelet activation and thrombus formation: a promising application to stop bleeding
Source: Clin Sci (Lond). 2025 Dec 18;139(24):1643–57. doi: 10.1042/CS20257213 (PMC12794321; doi:10.1042/CS20257213)
Supplement: online supplementary figure 1. [file cs-139-24-CS20257213-s001.pdf]

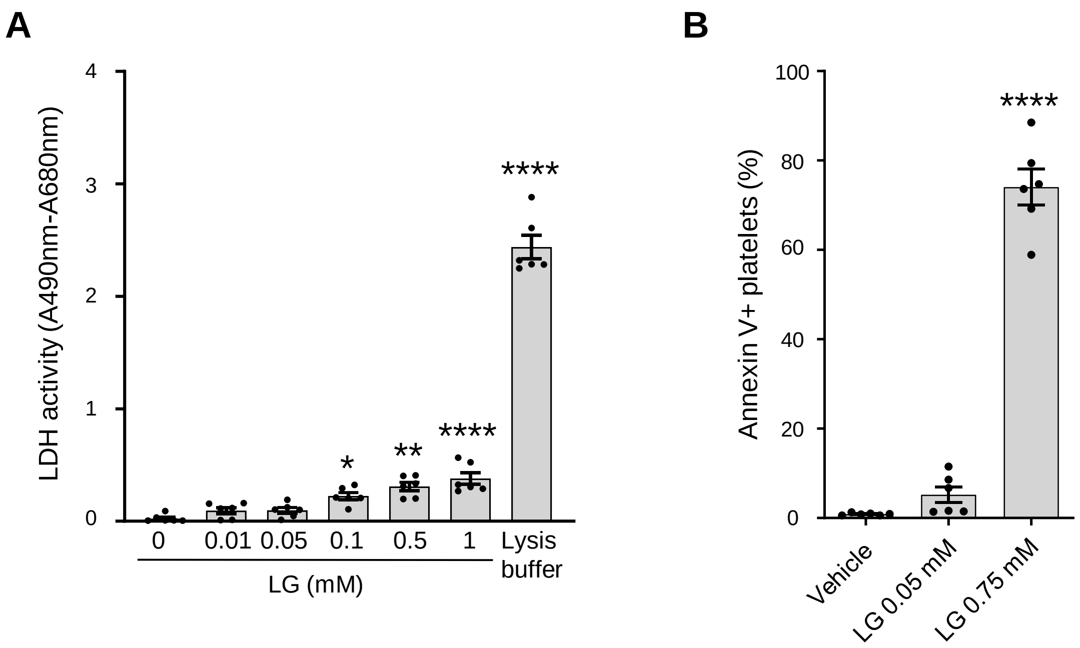

Supplementary figure 1: Effect of LG on LDH leakage and PS exposure. (A) Washed platelets from healthy donors were treated with increasing concentrations of LG for 10 minutes at 37°C. Thereafter, platelets were pelleted by centrifugation at 1800x*g* for 3 minutes and supernatants were used to detect LDH release by colorimetric method (Thermofischer Scientific), according to the manufacturer's protocol. Maximal LDH leakage was obtained with lysis buffer. B) Phosphatidylserine exposure was measured with annexin V binding by flow cytometry after 10 minutes of LG stimulation. Results, are expressed as LDH activity or percentage of annexin V positive platelets and are mean ± standard error of the mean (SEM) of 6 independent experiments (*p<0.05; **p<0.01; ****p<0.0001 according to one way ANOVA followed by Sidak's multiple comparisons test).
