# Supplementary figures and images for: Lauryl gallate promotes platelet activation and thrombus formation: a promising application to stop bleeding
Source: Clin Sci (Lond). 2025 Dec 18;139(24):1643–57. doi: 10.1042/CS20257213 (PMC12794321; doi:10.1042/CS20257213)

**A**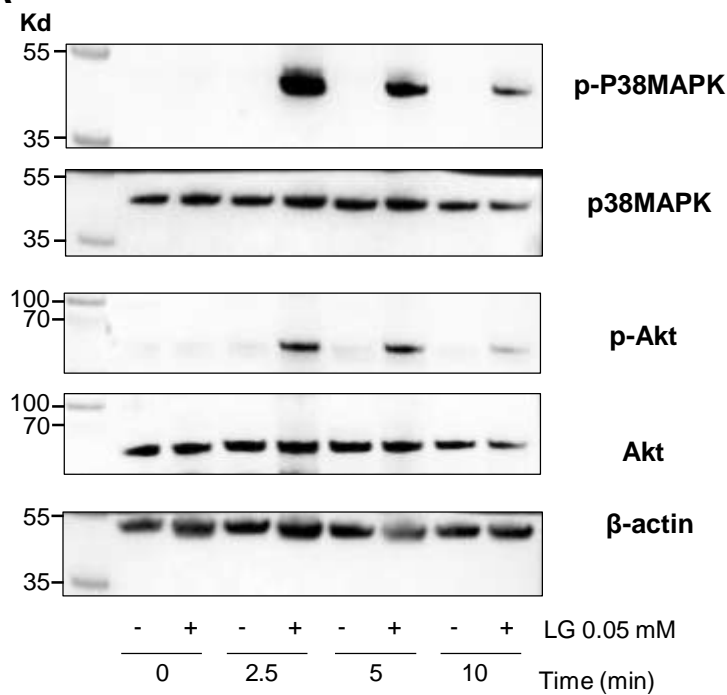**B**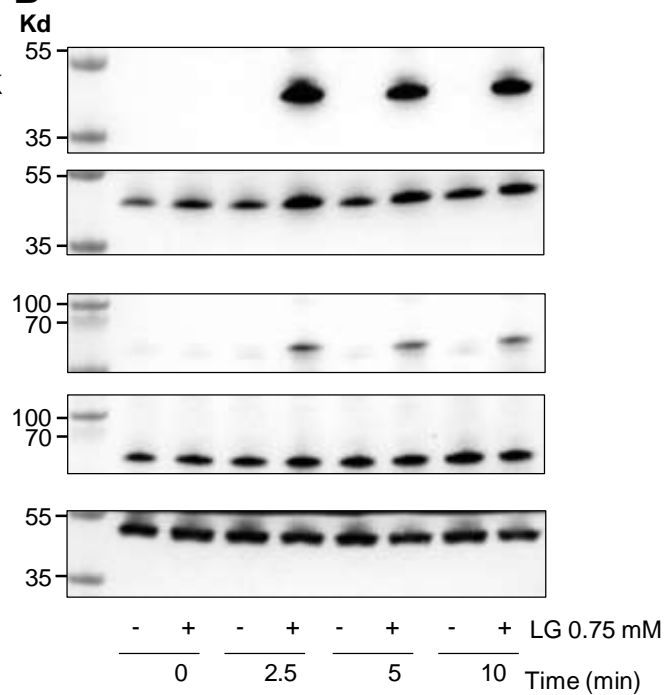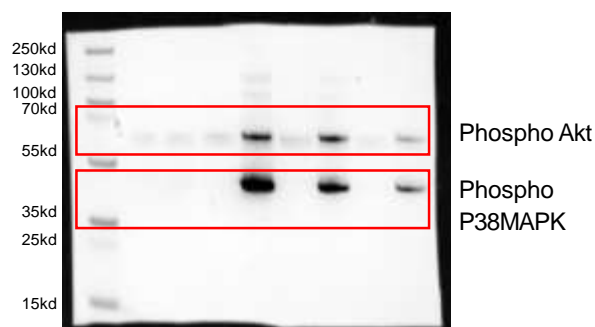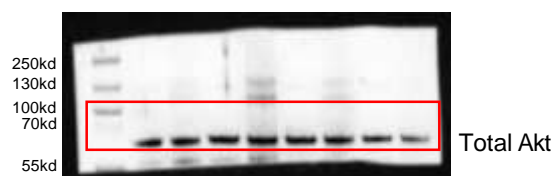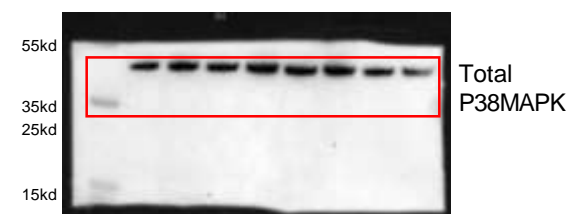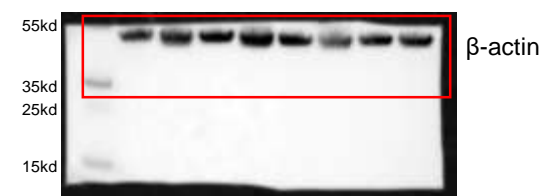

- + - + - + - + LG 0.05 mM  
0 2.5 5 10 Time (min)

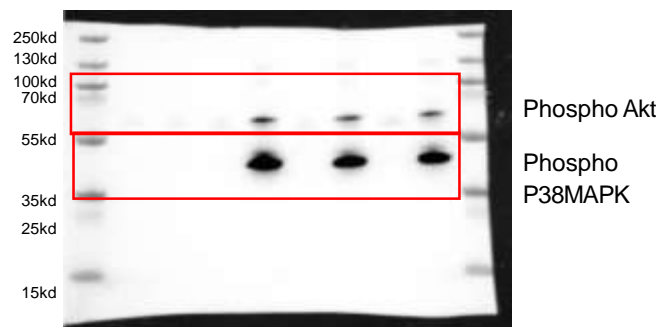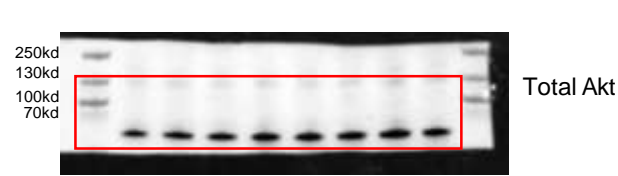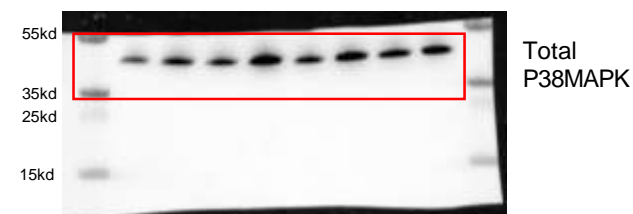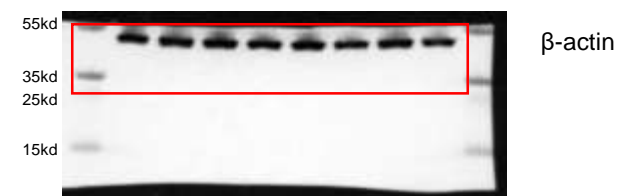

- + - + - + - + LG 0.75 mM  
0 2.5 5 10 Time (min)

Supplement: Uncited online supplementary figure 2. [file cs-139-24-CS20257213-s002.pdf]
